# Essential Oils; Their Application in Dentistry

**Published:** 1902-09-15

**Authors:** David Stern


### ESSENTIAL OILS; THEIR APPLICATION IN DENTISTRY.

BY DAVID STERN, B.S., D.D.S.

Read before the Cincinnati Odontological Society, February 28, 1902.

In his paper on putrescent pulps (*Dental Digest*, June, 1900), Dr. A. H. Peck says: "I would that we could all be first-class chemists, both analytical and synthetic, for then we could subject our medicines to the necessary tests and analyses, to determine whether or not they are best suited to successfully combat the various conditions to which they are being applied." The fact that the action of many drugs, especially of the essential oils, used in dentistry has puzzled all the investigators of these misterious bodies is probably due to the similarity in chemical construction. The literature on this subject, not very extensive as far as dentistry is concerned, is furnished by a few able men who have written scientifically. I have taken great liberties with the writings of Drs. Black, Peck, Burchard and Harlan, all of whom have devoted much time and care to the study of the subject of to-night's paper.

Being nearly alike in chemical reactions, the essential oils are distinguished chiefly by their physical properties. They consist mostly of terpenes and their close derivatives and are of the class of hydrocarbons, having the common formula $C_{10} H_{16}$, of which turpentine oil is the most familiar representative. They are disposed to absorb oxygen from the air and pass into camphors, two atoms of hydrogen being given off for each atom of oxygen taken on. This oxygen, in connection with moisture and heat, is again given off in the nascent state, and it is probable that it is the nascent oxygen which has the antiseptic and disinfecting action, as it chemically destroys septic matter. By "disinfection" I mean the destruction of living germs of disease, that is, disinfection is accomplished by the use of germicides.

In the practice of medicine the essential oils are not considered of practical importance as disinfectants, although experiments have been made with pathogenic bacteria. The results obtained with one—the bacillus of typhoid fever—show the time required by various oils to kill the microbe:

| | |
|---|---|
| Oil of cinnamon, of Ceylon . . | 12 minutes, |
| Oil of cloves . . . . . . . . | 25 minutes, |
| Oil of thyme . . . . . . . . | 35 minutes, |
| Thymol . . . . . . . . . | 35 minutes, |

while the oil of turpentine required more than twenty-four hours, and peppermint, eucalyptus, cajeput, wintergreen, and camphor took more than six days.

In dentistry we apply many other disinfectants and antiseptics, such as heat, hydrogen peroxid, permanganate of potash, bichlorid of mercury, etc., but the essential oils are considered of advantage as dressings for pulpless teeth, abcesses, putrescent pulps, etc., being readily applied to the diseased part and left there until the dentist sees the patient again. The important point is, with which of the oils are the best results obtained?

Dr. Burchard states that the essential oils act as protoplasmic poisons, without coagulating albuminious matter. They probably differ in germidical power, the oil of cassia leading in this respect, the oils of cloves, eugenol ($C_{10}H_{12}O_2$), which is the active principle of oil of cloves, eucalyptus, gaultheria, etc., following in the order mentioned. With the exception of the oil of peppermint, which has a cooling effect, they have a tendency to produce a warm and in some cases a burning sensation when applied in the mouth.

Dr. Peck has given us the results of investigation made in his bacteriological laboratory. To determine the irritating or non-irritating properties of these oils, he made a large number of experiments on his own person and also in connection with sores artificially produced on guinea pigs. He found that while the oil of cassia stands at the head of the essential oils as an antiseptic and is an excellent germicide when applied to suppurating surfaces, it is most poisonous and irritating in its effects upon soft tissue. On account of these characteristicts it is not recommended as a dressing in the root canals of teeth. It also has the tendency to discolor the teeth, and this discoloration is difficult to overcome. There is a difference between the oil of cassia of China and the oil of cinnamon, which is a product of Ceylon. It has been shown that the oil of cinnamon is not so irritating to the soft tissue as the oil of cassia.

The oil of cloves, consisting of caryophillin ($C_{10}H_{16}$) and the oxygen oil eugenol, it is non-irritating to soft tissue, leaves no discoloration and is effective in destroying microbes. It is therefore recommended for general use in the treating of pulpless teeth.

The oil of eucalyptus possessess more powerful antiseptic properties than phenol (carbolic acid), but it is not so irritating. Faust and Homeyer from the commercial oil obtained sixty percent terpene ($C_{10}H_{16}$), thirty percent thymol ($C_{10}H_{14}$) and the remainder an oxygenated compound ($C_{10}H_{16}O$) which they named eucalyptol. On account of its non-irritating qualities it is a splendid agent to place in root canals after the removal of the pulp.

Oil of wintergreen is a subject of disagreement between investigators, one holding that it is an excellent antiseptic, another, that it is useless in restraining the development of bacteria. It is composed principally of methyl salicylate ($CH_3C_7H_5O_3$) and does not belong to the class of terpenes previously mentioned. It is, however, surely a good agent to disguise the taste and odor of other drugs.

There are undoubtedly many others of the essential oils which have curative properties, but those which we have enumerated are the popular ones of dental practice. Although not an essential oil, there is a carbohydrate which is used in treatments as freely as all of the essential oils combined. It is phenol, the so-called carbolic acid, which in a concentrated solution (ninety-five percent) is a powerful escharotic on soft tissue, and when applied to the dental pulp forms a strong coagulum, but it is not considered an important antiseptic. It is in connection with some of the essential oils that its general popularity exists, and there is no remedy which is more liked by the profession than Black's "1-2-3."

The irritating effect of the cassia seems to be modified by the mildness of the wintergreen, and the coagulating power of the phenol does not appear to be as great in the presence of the cassia and wintergreen. It seems that by the combination of the three in the prescribed proportions a new chemical union is formed which does not posses the properties of the individual factor and is highly satisfactory in the treatment of diseased tissue.

The fact that often the oils do not give the favorable results anticipated may in a measure be due to the adulteration of the drugs. Especially are those from the Orient liberally treated with alcohol and domestic drugs, and few of them will stand an analytical investigation.

DISCUSSION.

Dr. H. A. Smith, Cincinnati: An antiseptic, if we take carbolic acid as an example, depends upon its soluble action. The essential oils are only slightly soluble, and for this reason they are preferable, because the antiseptic quality lasts longer. The potency of carbolic acid is soon destroyed by dilution. If the oils had perhaps one-half that potency I think they would be equally as good. The most soluble ones are effective for the shortest time.

Dr. J. S. Cassidy, Covington, Ky.: The subject of the essential oils has always been a fascinating one to me because of their complex nature, and because they all contain more or less terpenes. It is well known that these latter become oxidized and give up free oxygen and camphor. These oils also contain other bodies as well as terpenes, and I had thought of criticizing one remark the essayist made, but which he afterwards modified—that terpenes were the active principle of all these oils. From time immemorial oil of cloves has been a domestic antidote for toothache, the wise old women realizing it just as we do, without the sience. Years ago Dr. Hunter stated that all a dentist needed was arsenic and carbolic acid, a pound of one and a barrel of the other. If I were limited to the use of one of these oils I should select oil of cloves. The terpenes of these oils when oxidizing change their colors, darkening them, especially oil of cassia. Oil of cloves turns dark yellow, which action is due to a camphor, sometimes called a resinous matter, that is soluble in water. When I find the teeth discolored by this camphor I remove the stains with alcohol. I agree with Dr. Smith that carbolic acid is a poor antiseptic, losing its identity by solution in water. I have always felt that the health-giving quality of the atmosphere in the pine forests of Kentucky, North Carolina and elsewere was due to the setting free of the essential oils distilled by nature from these trees.

Dr. Smith: I would ask if the odor given off by the oils while oxidizing is antiseptic or beneficial in any sense?

Dr. Cassidy: The odors that come from bodies like musk bring to us part of the musk itself. If the odor comes from oil of cloves, it is still the oil containing the active principles of the terpenes and the eugenic acid, but that portion of the oil which develops into a resin of course prevents the bacteria from acting on the part effected. Oils which develop into acids, like oil of cassia, are more irritating to tissue than those containing alcohol. Dr. Peck has been very enthusiastic in investigating along these lines. When formaldehyd was brought out he applied a cloth saturated with it to his leg, so as to note the effect. Ulceration set in, and he was a very sick man for three weeks, no remedy that he tried being effective. Finally he applied pure oil of cloves, and the sore healed in few days.

Dr. A. G. Rose, Cincinnati: Is it true that chloropercha will dissolve oil of eucalyptus?

Dr. O. N. Heise, Cincinnati: I have always advocated the use of essential oils in the treatment of pulpless teeth and putrescent pulps, but the idea that you do not want to use anything which coagulates is a mistake, as a coagulant at times is necessary to give nature a chance to forget herself, so to speak.

Dr. Stern: In replying to Dr. Rose, gutta-percha is soluble in oil of eucalyptus. Replying to Dr. Heise, coagulation is oftentimes needed, and I have no idea of depreciating the use of carbolic acid. The greatest difficulty in making these experiments in the fact that we can not always secure pure chemicals.—*Digest*.

